# Chitosan-Based Intranasal Vaccine against *Escherichia coli* O157:H7

**DOI:** 10.7508/ibj.2016.02.005

**Published:** 2016-04

**Authors:** Tahere Doavi, Seyed Latif Mousavi, Mehdi Kamali, Jafar Amani, Mahdi Fasihi Ramandi

**Affiliations:** 1 Dept. of Biology, Faculty of Basic Sciences, Shahed University, Tehran, Iran;; 2 Nano Biotechnology Research Center, Baqiyatallah University of Medical Science, Tehran, Iran;; 3 Applied Microbiology Research Center, Baqiyatallah University of Medical Science, Tehran, Iran;; 4 Molecular Biology Research Center, Baqiyatallah University of Medical Science, Tehran, Iran

**Keywords:** *EnterohemorrhagicEscherichia coli*, Nanoparticles, Intranasal vaccination

## Abstract

**Background:**

*Enterohemorrhagic* *Escherichia coli* (EHEC) O157:H7 is an infectious zoonotic pathogen causing human infections. These infections, in some cases, can lead to hemolytic uremic syndrome and its life-threatening complications and even death worldwide. The first intimate bacterial adhesion, intimin (I), with its own receptor translocated intimin receptor (Tir) and* E. coli* secreted protein A, acting as Tir conduit, are highly immunogenic proteins for vaccine development against *E. coli* O157:H7.

**Methods:**

A chimeric trivalent recombinant protein was previously found to be a suitable strategy for developing vaccines against *E. coli* O157:H7. In this study, the recombinant EIT (rEIT) was used to design a protective EHEC nasal nanovaccine. Chitosan and its water-soluble derivative, trimethylated chitosan (TMC), as muco-adhesive biopolymers, are good candidates for preparation of nanovaccines.  Using the electrospraying technique, as a novel method, we could obtain particles of rEIT loaded with chitosan and TMC on a nanometer scale. Mice were immunized with intranasal administration or intrapretoneal injection of rEIT.

**Results:**

The rEIT-specific immune responses (IgG and IgA) were measured by indirect ELISA. Only nasal administration of chitosan electrospray and TMC formulation produced significant secretion IgA. Intranasal administration of nanovaccine reduced the duration of bacterial fecal shedding on mice challenged with *E. coli* O157:H7.

**Conclusion:**

Since development of mucosal vaccines for the prevention of infectious diseases requires efficient antigen delivery; therefore, this research could be a new strategy for developing vaccine against *E. coli* O157:H7.

## INTRODUCTION

*Enterohemorrhagic Escherichia coli *(EHEC) O157:H7 is an important foodborne pathogen and the causative agent of large outbreaks of hemorrhagic colitis and sporadic cases of serious systemic microangiopathy such as hemolytic uremic syndrome and thrombotic thrombocytopenia purpura^[^^1^^]^. The vaccination of healthy cattle, as reservoirs of *E. coli *O157:H7, is of importance, which in turn prevent the transmission of infection to human beings.^[^^2^^]^. 

Several virulence factors encoded by a pathogenicity island, known as the locus of enterocyte effacement (LEE), enable EHEC to colonize and form characteristic of attaching and effacing lesions in mammalian cells^[^^3^^]^. Some putative virulence factors and other translocating effector proteins secreted using the LEE-encoded type III secretion system mediate an essential process for EHEC pathogenesis. These essential virulence factors include EspA (*E. coli* secreted protein A), EspB, EspD, and Tir (translocated intimin receptor) ^[^^4^^]^. Type III secretion system translocator protein EspA induces a filamentous structure that forms an essential bridge for translocation of EspB, EspD, and intimin receptor (i.e. Tir) into the host cells. Intimin is defined as the primary adhesion factor of EHEC that is encoded by a part of LEE, called *eae* gene^[^^5^^]^. Intimate bacterial attachment to the host cell, mediated by Tir translocation via EspA translocator filaments and following intimin-Tir interactions^[^^6^^]^. In our previous studies^[^^7^^-^^9^^]^, the immunogenic properties of a chimeric recombinant EIT (rEIT) protein, including EspA, intimin, and Tir were studied.

Intranasal administration is considered as one of the promising routes for needle-free antigen/protein delivering in novel vaccination strategies^[^^10^^]^. Intranasal administeration offers many advantages as low enzymatic activity of nasal cavity and thus the reduction of the necessary antigen dose and more acceptable alternative for patients^[^^10^^,^^11^^]^. The intranasal administration of EHEC vaccine has proved to be a very effective and additional protection correlated with SIgA^[^^12^^-^^14^^]^. A nasal delivery route can effectively induce both the humoral and mucosal immune responses^[^^15^^]^.

The presence of about 400 microvilli per nasal epithelial cell provides a large surface area for the nasal mucosa and can be a suitable nasal cavity for mucosal immunization^[^^16^^]^. Harsh mucosal environment of nasal cavity can reduce vaccine absorption across the nasal mucous membranes^[^^17^^]^. Therefore, effective adjuvants and delivery systems are needed to potentiate vaccine immunogenicity and to protect their antigens from the harsh nasal mucosal environment^[^^18^^]^. 

Biocompatible and biodegradable chitosan is a muco-adhesive biopolymer with absorption-enhancing property to improve immunogenicity of intranasal antigen delivery systems^[^^19^^]^. Due to their properties such as opening up the tight junctions between epithelial cells, muco-adhesive natural polymers have attracted great attention in the design of intranasal vaccine delivery of subunit antigen or peptides^[^^19^^]^. Chitosan and its water-soluble derivative, trimethylated chitosan (TMC)^[^^20^^]^, are suitable candidates for preparation of intranasal nanovaccines^[^^21^^]^. Chitosan micro/nanoparticles can be easily fabricated via the electrospraying method using electrical fields^[^^22^^]^. Electrospraying is a rapid, simple and affordable method for polymeric micro/nanoparticle fabrication^[^^23^^]^. In this research, we employed the electrospraying technique to obtain particles of rEIT loaded with chitosan and TMC on a nanometer scale. Mice were immunized with intranasal administration or intrapretoneal injection of rEIT, and the specific immune responses (IgG and IgA) were measured by indirect ELISA. Intranasal administration of nano-vaccine reduced the bacterial fecal shedding on mice challenged with *E. coli *O157:H7.

## MATERIAL AND METHODS

**Bacterial strain**

EHEC serotype O157:H7 (strain ATCC 35218) and the previously constructed rEIT in pET28a plasmid were available in our laboratory^ [^^7^^]^. The recombinant and wild-type EHEC strains were grown in Luria-Bertani (LB) broth, supplemented with 40 µg/ml kanamycin (Sigma, Germany) as required. *E. coli* isolates were stored in a LB broth containing 20% glycerol at -70ºC.

**Expression and purification of recombinant EIT protein**

Expression of the synthetic *EIT* gene was performed in *E. coli *BL21 (DE3*)*. The rEIT, which was purified with Ni-NTA (Qiagen, Germany) resin under native conditions, was verified on 10% SDS-PAGE^[^^7^^]^.

**Synthesis of trimethylated chitosan polymer**

TMC polymer was prepared by reductive methylation procedure of chitosan based on the reductive methylation reactions between chitosan and methyl iodide. Also, 2 g chitosan, 4.8 g sodium iodide, and 10 ml 20% (w/v) aqueous NaOH were dissolved in 80 ml 1-methyl-2-pyrrolidinone on water bath at 60ºC for 20 min. Subsequently, 10 ml methyl iodide was added, and the resulting mixture was kept in a Liebig condenser for 30 min. Next, 10 ml methyl iodide and 10 ml 20% (w/v) aqueous NaOH were added to the mixture and was incubated in a similar Liebig condenser at a controlled temperature of 60ºC for 30 min. In order to precipitate the product, 300 ml diethyl ether was sequentially added three times to the solution mixture. The product was dissolved in 100 ml 10% NaCl aqueous solution to exchange the iodide ion with chloride. The final product obtained from centrifugation was freeze-dried and kept in darkness^[^^24^^]^. TMC powder was dissolved in double distilled water to 1 mg/ml concentration (pH 7.4) and stored at -20ºC for further use.

**Preparation of chitosan solution for electrospraying**

For hydrolyzation of chitosan chains, a 1.25% (w/v) chitosan mixture was prepared in 50% (V/V) NaOH and incubated at 95°C for 48 h. The mixture was then filtered and washed with double distilled water. After several washing steps by glacial acetic acid, the chitosan was dried at 60°C for 16 h. A chitosan solution of 7.5% (w/v) prepared from processed chitosan in 70% (v/v) acetic acid was incubated under mild shaking at 25°C for 18 h. The chitosan was then dissolved in acetic acid to obtain a homogenous chitosan solution^[^^25^^]^. rEIT (0.1 mg/ml) and chitosan solution (volume ratio 1:1) were mixed at the final antigen concentration of 20 µg/ml. The mixture containing chitosan and antigen solution was used for electrospraying to produce the nanoparticles.

**Generation of nanoparticles with electrospraying**

rEIT-chitosan and rEIT-TMC solutions were placed separately in 3-ml electrospraying syringe with a metallic needle of 0.65 mm internal diameter. A 20 kV current was applied for electrospraying. The distance between needle tip and collector was optimized at 10 cm, and feeding rate was adjusted to 1 ml/h^[^^26^^]^. rEIT solutions (1 mg/ml) were combined in a volume at the ratio of 1:1 with each of the chitosan and TMC solutions at final mixture concentrations of 20 µg/ml and then electrosprayed as described above.

**Morphological properties, particle size and the surface charge of particles**

The morphological properties were examined using a Philips-XL30 scanning electron microscope (SEM). For this purpose, chitosan and TMC electrosprayed particles along with rEIT antigen were coated with a thin layer of gold and examined under Philips-XL30 SEM at an 22 kV accelerating voltage. Particle size and zeta potential of electrosprayed chitosan and TMC particles along with rEIT were measured by laser diffraction using a Zetasizer (Malvern, UK)^[^^27^^]^.

**Animal immunization**

Female five- to six-week-old BALB/C mice (n=25) procured from Pasteur Institute of Iran were divided into two tests (T1, T2) and three control groups (C1, C2, and C3). Each mouse in the T1 group was immunized directly by nasal administration of 20 μg *electrosprayed* rEIT protein along with chitosan (Sigma, Germany). T2 group received 20 μg of *electrosprayed* rEIT protein along with TMC (volume ratio 1:1). C1 group was injected intraperitoneally with 20 μg rEIT along with complete Freund's adjuvant (Sigma, Germany) in the first injection and incomplete Freund's adjuvant (Sigma, Germany ) in two subsequent injections, served as positive control. C2 group was administered intranasally with only chitosan served as negative control. Each mouse in C3 group was administered through the nasal route with 20 μg of non-electrosprayed rEIT protein with chitosan as an adjuvant. The description of groups is shown in Table 1. All groups received their related formulations at the same time on days 0, 28, and 42^[^^9^^,^^20^^,^^28^^]^.

**Table 1.** Immunization administered to the groups

**Experimental group **	**Route of administration**	**Administrated formulation**	**Immunized schedule**		**Serum sampling day**	**Antigen dose (µg/mice)**
			**Dose**	**Day**	**Days after administration**	
Test 1 (T1) immunized mice	Intranasal	electrosprayed chitosan-rEIT nanoparticles	first	0	14	20
			second	28	7	20
			third	42	7	20
Test 2 (T2) immunized mice	Intranasal	electrosprayed TMC-rEIT nanoparticles	first	0	14	20
			second	28	7	20
			third	42	7	20
Control 3 (C3) immunized mice	intrapretoneal	rEIT-CFA	first	0	14	20
		rEIT-IFA	second	28	7	20
		rEIT-IFA	third	42	7	20
Control 2 (C2) non-immunized mice	Intranasal	non-electrosprayed chitosan-rEIT	first	0	14	20
			second	28	7	20
			third	42	7	20
Control 1 (C1) non-immunized mice	Intranasal	alone chitosan	first	0	14	0
			second	28	7	0
			third	42	7	0

CFA, Complete Freund's adjuvant; IFA, Incomplete Freund's adjuvant; recombinant EIT (rEIT)

**Sample collection**

Blood samples were collected retro-orbitally after each immunization. The sera were stored at -70°C for further analysis. In order to assess SIgA titers, mice fecal and eye wash samples were collected weekly after each immunization^[^^29^^]^. Fecal samples (1 g) from each mice group were mixed immediately with 500 µl PBS containing 0.05% (w/v) sodium azide and 10 µl/mg protease inhibitor cocktail (Roche, Switzerland) and kept at 4°C overnight. After centrifugation at 20,000 ×g at 4°C for 10 min, the supernatants were stored at -70°C. Eye wash samples were collected by washing the eyes with 100 µl (50 µl for each eye) of cool sterile PBS containing protease inhibitor, and the samples were immediately frozen at -70°C^[^^30^^]^.

**Antibody responses of serum, feces, and eye wash**

Specific antibody responses in serum, feces, and eye wash samples were evaluated by an ELISA. Also, 96-well plates (Nunc, Denmark) were coated with 5 µg the purified rEIT protein diluted in a coating buffer (64 mM Na_2_CO_3_ and 136 mM NaHCO_3_, pH 9.8)^[^^9^^]^. The plates were washed three times with PBS containing 0.05% Tween 20 (PBS-T) and blocked with 3% gelatin in PBST and incubated at 37°C for 1 h. For determination of the relative IgG and IgA titres, sera samples were serially diluted in PBS-T from 1:500 to 1:512,000 for IgG and from 1:20 to 1:10240 for IgA whereas fecal and eye wash samples were serially diluted in PBS-T from 1:20 to 1:10240. The diluted samples were added to ELISA plates and incubated at 37ºC for 1 h. After washing in PBS-T, the plates were added with a 1:5000 dilution of horseradish peroxidase goat anti-mouse IgG (Sigma, Germany) or horseradish peroxidase goat anti-mouse IgA (1:10,000 in PBS-T) (Sigma, Germany), followed by incubation at 37ºC for 1 h. The wells were then washed three times in PBS-T. Finally, 100 µl 3,3',5,5'-tetramethylbenzidine (Sigma, USA) was added to each well and incubated at room temperature for 15 min. The reaction was stopped by the addition of 2 M H_2_SO_4_, and the absorbance was read at OD_450_ on a microplate reader (BioRad, USA). 

**Animal study**

Fourteen days after the last immunization, the mice were orally challenged with a lethal dose of *E. coli* O157:H7 (ATCC: 35218). Two days prior to the challenge, all mice groups were pretreated with drinking water containing streptomycin sulfate (5 mg/ml) to decrease the normal gut bacterial flora. The streptomycin-treated mice were fasted overnight and subsequently fed with 10^10^ CFU of *E. coli* O157:H7. The mice fecal samples were collected and weighted daily for three weeks. Fecal samples (approximately 0.1 g) were suspended in 1 ml LB broth and incubated at room temperature for 2-4 h to allow the fecal pellets to be soften. The fecal mixture was then vortexed to homogeneity, followed by plating onto Sorbitol MacConkey agar plates containing tellurite. The plates were incubated at 37ºC overnight, and *E. coli* O157:H7 colonies were counted^[^^7^^]^. 

**Bacterial adhesion and growth inhibitory effect**

Caco-2 cells (Pasteur Institute of Iran, Tehran) were grown in a culture flask containing RPMI medium and 10% FBS. The confluent cells attached to the culture flask were trypsinized and distributed on a sterile round coverslip placed on the bottom of the 24-well culture plates and incubated in 5% CO_2_ at 37ºC for 48 h. An overnight culture of EHEC cells grown to exponential growth phase was washed three times with PBS and adjusted to an OD: 0.6 at 600 nm (3.5×10^8^ of *E. coli* O157:H7). Antisera (150 µl) from intranasally immunized mice (T1 and T2 groups) were added to the 300 µl bacterial suspension and incubated at room temperature for 20 min. The Caco-2 cell line treated with unimmunized mice antisera served as a negative control. The bacterial mixture was added thoroughly to monolayer Caco-2 cells and incubated at room temperature for 1 h^[^7^]^. The monolayer Caco-2 cells were trypsinized after washing three times with PBS. Aliquots of the serial dilutions of Caco-2 supernatants were plated on LB agar plates and incubated at 37ºC overnight. Colonies were counted to determine the total CFU/ml and the percent bacterial adhesion of each sample. The mean number of adherent bacteria per cell was determined by the measurement of Caco-2 cells on each coverslip^[^^31^^]^. 

**Histopathological studies**

Eight days after oral challenge, the mice were bled for sera collection and sacrificed by cervical dislocation. Colon and caecum of each mouse were photographed and dissected for histological examinations. Specimens were plated in glass dishes, followed by rapid fixation in 10% formaldehyde. The tissue sections were embedded in paraffin and stained with hematoxylin and eosin and examined under a light microscope. 

**Statistical analysis**

Means±standard deviations (SD) of three independent experiments were performed. The statistical analyses were carried out with a SPSS statistical software (version 20). Bonferroni's multiple comparisons test was applied to compare the test and control groups in serum antibody determination and fecal shedding of bacteria. Analysis for the significance differences of EHEC attachment inhibition on Caco-2 cells was also tested using Bonferroni multiple comparison test. The *P*<0.05 was considered statistically significant.

**Fig. 1:**
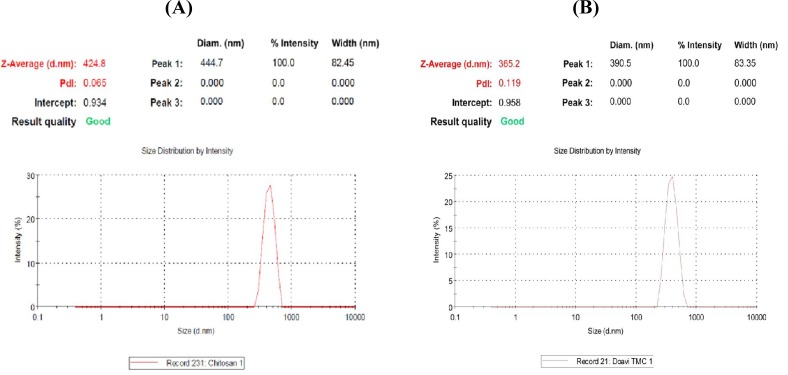
The nanoparticles size distribution measured by Zetasizer. A) Electrosprayed chitosan nanoparticles containing rEIT (about 424/8 nm); B) Electrosprayed TMC nanoparticles containing rEIT (about 365/2 nm).

## RESULTS

**Morphological properties, particle size, and surface charge of electrosprayed particles**

**Fig. 2:**
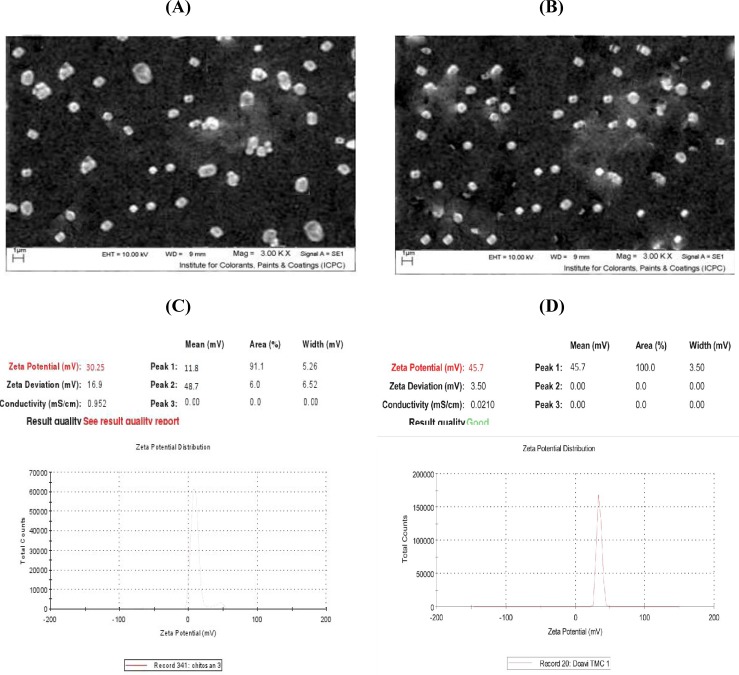
SEM images of electrosprayed nanoparticles and zeta potentials. A) SEM images of electrosprayed chitosan nanoparticles containing rEIT; B) TMC nanoparticles containing rEIT; C) Zeta potential of electrosprayed chitosan nanoparticles containing rEIT (about +30/25 mV); D) electrosprayed TMC nanoparticles containing rEIT (about +45/7 mV).

The particle size and zeta potential results are shown in Figures 1 and 2D, respectively as well as in Table 2. The size of electrosprayed chitosan particles along with rEIT was about 424.8 nm with a polydispersity index of about 0.065 (Fig. 1A) and that of TMC along with rEIT was about 365.2 nm with a polydispersity index of 0.119 (Fig. 1B). The surface charge of chitosan and TMC nanoparticles was measured by zeta potential measurement (Fig. 2C and 2D). The zeta potential values of the chitosan and TMC nanoparticles were determined to be +30/25 mV and +45/7 mV, respectively. The positive values of nanoparticles zeta potential indicated that the chitosan and TMC nanoparticles possessed positive surface charges. The spherical morphology of nanoparticles was monitored using SEM (Fig. 2A and 2B).

**Determination of antibody responses to rEIT**

**Fig. 3:**
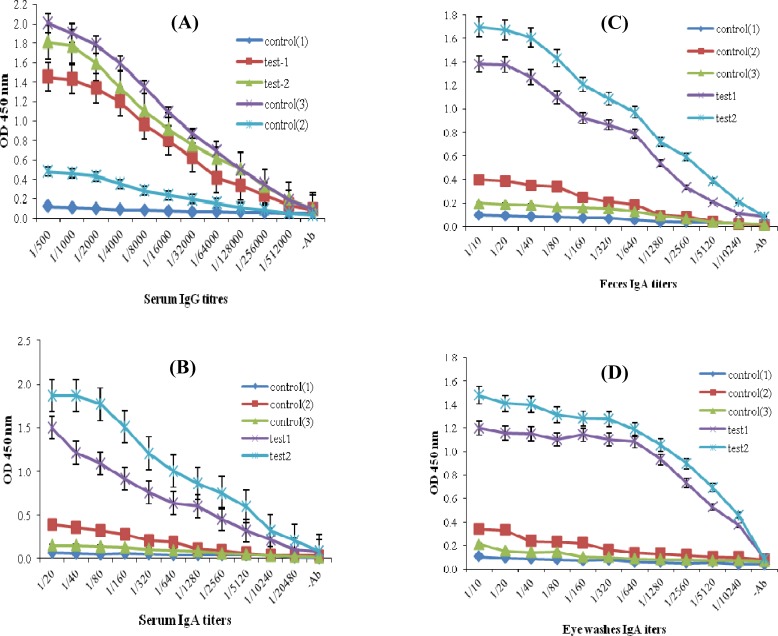
EIT-specific IgG and IgA. Test 1, intranasal immunization with electrosprayed nanoparticles chitosan-rEIT; Test 2, intranasal immunization with electrospraed TMC-rEIT nanoparticles; Control 1, intraperitoneally immunization with rEIT adjuvanted by Freund's adjuvant; Control 2, non-immunized mice nasally administered with chitosan alone; Control 3, non-immunized mice nasally administered with non-electrosprayed chitosan-rEIT. Mice immunization was conducted three times during 42 days. The sera, fecal, and eye wash samples were collected after each immunization and assayed for rEIT-specific responses, including (A) serum IgG titers, (B) serum IgA titers, (C) fecal IgA titers, (D) eye washes IgA titers using an ELISA method. Non-immunized mice and intraperitoneally immunized samples were used as negative and positive control, respectively (*P*<0.05).

**Fig. 4:**
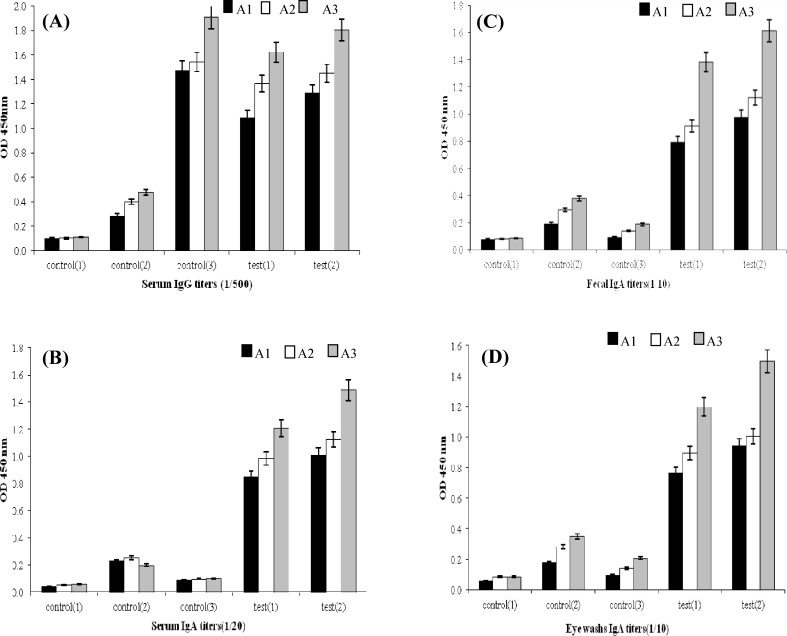
Comparison of inducted EIT-specific IgG and IgA antibody titration. A and B, IgG titration; C and D, IgA titration. (A1, A2, and A3 in curves show first, second, and third intranasal booster immunizations of mice with electrosprayed nanoparticles chitosan rEIT for test and control groups

Titration of serum samples from intranasally mice immunized with purified rEIT compared to the C1 and C2 groups showed a significant EIT-specific IgG and IgA antibody responses. The titers of anti-EIT-specifice IgG antibody were clearly detectable even at 1:512000 dilution (Fig. 3A). Also, the titers of EIT-specific IgA antiserum in two intranasally immunized mice groups were detected up to 1:20480 titers (Fig. 3B) while no significant difference (*P*=0.17) was found in IgG antiserum titres in intranasally immunized mice (T1 and T2) compared to the control group (C3) intraperitoneally immunized with rEIT (Fig. 3A). The mice immunized intranasally with rEIT showed significant titers of EIT-specific SIgA antibody (up to 1:10240 dilution) in fecal and eye wash samples compared to the control groups (Fig. 3C and 3D). No antibody SIgA response was detected in the fecal and eye wash samples of intraperitoneally immunized mice (Fig. 3C and 3D). The data in Figure 4A and 4D demonstrated that the elicitation of a higher anti-EIT IgG and IgA could be achieved after the third immunization using an intranasal nano-administration. The presence of rEIT purified protein was verified on 10% SDS-PAGE as the only band in all analyzed eluted fractions of rEIT purification process (Fig. 5). 

**Bacterial adhesion and growth inhibitory effect**

Colony count of Caco-2 cell supernatant serial dilutions plated in LB agar indicated 99% bacterial adhesion to cell lines when treated by non-immunized mice sera. The agar plating assay of EHEC cells pretreated with intranasal immunized mice antisera showed 65% blocking of their adherence to Caco-2 cells (Table 3). 

**Table 2:** The mean of chitosan and TMC particle size and zeta potential

**Sample**	**Solution concentration (µg/ml)**	**Mean particle** **size (nm)**	**PDI**	**Zeta potential (mV)**
Chitosan particles + rEIT	20	424.8	0.065	+30/25
TMC particles + rEIT	20	365/2	0 .119	+45/7

PDI, polydispersity index; recombinant EIT (rEIT)

**Animal challenge with** ***E. coli*** **O157:H7**

**Fig. 6:**
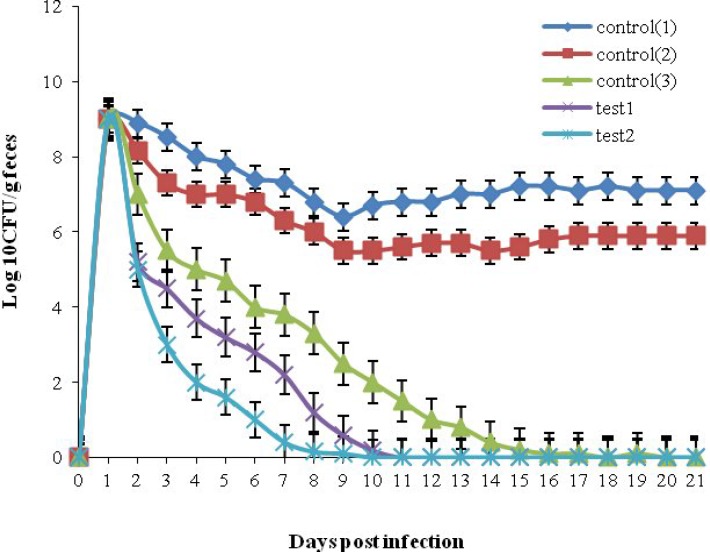
*E. coli *O157:H7 shedding following intranasal vaccination compared to intrapretoneal immunization. Mice in test or control groups were orally gavaged with 10^10^ CFU of *E. coli* O157:H7, and bacterial fecal shedding of each mouse was detected during three weeks. The reliable detection limit of plating was 100 CFU/0.1 mg fecal samples. Test 1, intranasal immunized mice with electrosprayed nanoparticles chitosan rEIT; Test 2, intranasal immunized mice with electrosprayed TMC-rEIT nanoparticles; Control 1, mice administrated with chitosan alone; Control 2, mice administrated with non-electrosprayed chitosan-rEIT; Control 3, intraperitoneally immunized mice with rEIT adjuvanted by Freund's adjuvants; (*P*<0.05).

Unimmunized control mice groups (C1 and C2) showed high levels (10^8^ CFU) of bacterial shedding in their feces during the three-week sampling period. The time and level of shedding in all immunized mice were declined gradually in T1, T2, and C3 groups and stopped after 8, 9, and 10 days, respectively (Fig. 6). The level of bacterial shedding between three immunized groups was not significant (Fig. 6) whereas the reduction of bacterial shedding in T1, and T2, and C3 groups was statistically significant (*P*<0.05) as compared to the unimmunized C1 and C2 groups.

**Histological examination**

**Fig. 7:**
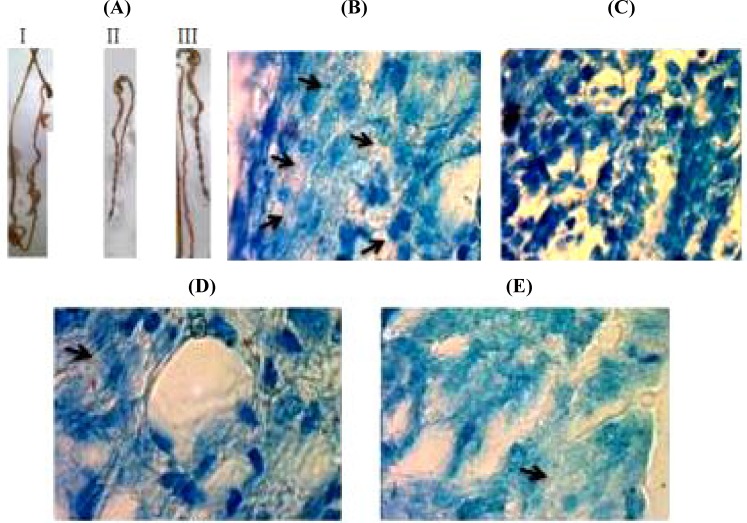
Caecum and colons micrograph images of infected mice after oral challenge with *E. coli *O157:H7. A) I: The deformed colon and caecum of unimmunized mice, II: Normal colon and caecum of intranasally immunized infected mice exhibiting normal appearance and well-formed dark stools in the distal colon, III: Colon and caecums of uninfected mice were similar to those of intranasally immunized infected mice. B) Unimmunized infected mice present intimate bacterial adherence on caecum and colon cells (arrows); C) Intranasal immunized with electrosprayed nanoparticles TMC-rEIT showing smoothen surface epithelium without any bacterial attachment; D) Intranasally immunized mice with electrosprayed chitosan-rEIT nanoparticles; E) Intrapretoneal immunized mice with rEIT, which exhibited the reduction in bacterial adhesion

Microscopic observations of colon and caecum section are shown in Figure 7B, 7C, 7D, and 7E. The stained sections of mice immunized intranasally or intraperitoneally demonstrated a healthy caecum as of the uninfected mice in terms of shape and size exhibiting normal appearance and well-formed dark stools in the distal colon (Fig. 7A).

Unimmunized infected mice showed thickening shape and shortening of diameter in a distal colon with a few pale yellowish stool with diffused watery blood appearance. The colon weight of infected immunized mice was approximately equal to that of uninfected normal mice (<0.17 g). Also, the colon weight (0.40 g) of non-immunized infected mice was approximately double the normal (Fig. 7A). Based on the macroscopic observations of colons and caecum section, the number of bacteria in the intestinal cells of infected immunized mice was lower than that in unimmunized infected groups (Fig. 7B, 7C, 7D, and 7E). Notably, the bacterial number of intranasal immunized mice in T2 group was negative (Fig. 7C)

## DISCUSSION

Immunization with type III secreted proteins is one of the strategies to reduce the *E. coli *O157:H7 prevalence. *In cattle, EHEC* *O157:H7* *secreted protein vaccines* *has been shown to decrease the rate of* E. coli *O157:H7* *infections and significantly limit its prevalence. T*he recombinant construct containing trivalent immunogen EspA, intimin, and Tir has been indicated to have effective properties in induction of protective immunity and development of a vaccine against *E. coli *O157:H7^[^^7^^,^^9^^]^. Intranasal route, which is one of the most promising mucosal delivery route for the administration of vaccine^[^^10^^]^, induces mucosal immunity toward systematic immune responses and effectively imparts protection to many pathogens adhering to mucosal surfaces^[^^15^^]^. Furthermore, * E. coli* O157:H7 intranasal vaccine can be a more appropriate candidate than other mucosal vaccines due to its high accessibility and low proteolytic enzymatic activity that provides greater antigenic stability and lower doses of required antigens^[^^10^^,^^11^^]^. 

**Fig . 5:**
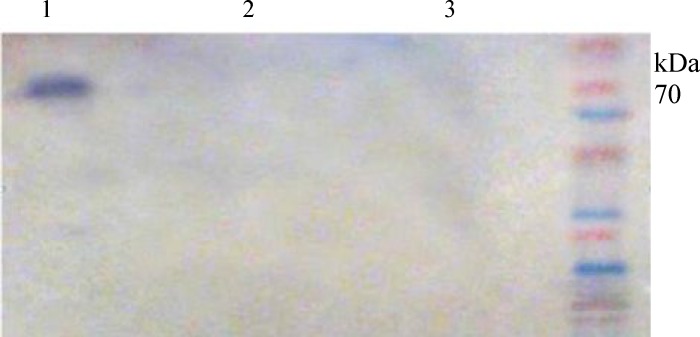
Western-blot analysis of rEIT-specific IgG antibody in intranasally immunized mice. Lane 1, sera from intranasally immunized mice; Lane 2, sera from unimmunized mice; Lane 3, protein weight marker.

In the present study, a constructed rEIT, including a truncated form of EspA (EspA 120), conserved carboxy-terminal region of intimin (intimin 282), and a fragment of Tir (residues 258-361, Tir 103) was applied to design a new intranasal nanovaccine. A plant-based oral vaccine (rEIT) used for mucosal delivery showed more efficacy with the combination of parenteral injection as a prime booster and an oral strategy^ [^^9^^]^. In contrast, our current rEIT intranasal vaccine demonstrated considerable titers of IgG-, IgA-, and sIgA-specific antibodies after the first nasal administration, followed by administrations without prime injection (Fig. 4). New findings of the current study also indicated that intranasally immunized mice significantly induce fecal and ocular sIgA compared to intrapretoneal immunized mice with subsequent decrease in the bacterial shedding after oral challenge (Fig. 6). 

Intranasal vaccines have several limitations, including rapid antigen residence time in nasal cavity and the inefficient permeability of administered antigens through the nasal epithelial barriers^[^^15^^]^.

**Table 3:** Inhibition of *E. coli* O157:H7 attachment to Caco-2 cells in intranasally immunized and non-immunized mice sera

**Test sera**	**Positive Caco-2 cells (%)** a	**Mean number adhesion** b
Non-immunized mice	99	13.6±0.66*
Intranasal immunized mice	34	2.1±0.03

* (*P*<0.05).The percentage of *E. coli *O157:H7 cells adhered to Caco-2 cells treated with intranasal immunized mice sera was significantly lower than that treated with non-immunized mice sera

a *The percentage* of bacterial counts *adhered* to *Caco-2 cells; *

b *the mean number of adherent bacteria cells per Caco-2 cell.*

Therefore, to overcome these limitations, strong antigen-adjuvant formulations and effective delivery systems are essentially required for the improvement of the nasal vaccine ^[^^18^^]^. Recently, the non-toxigenic polymeric nanoparticles, such as chitosan and its derivatives, with biocompatible and biodegradable properties are gaining attention as unique antigen delivery systems^[^^19^^,^^32^^]^. Nasal delivery of chitosan and TMC muco-adhesive particles can increase the cellular antigen uptake through M cells and nasal epithelial cells. This can lead to M-cells to targeting and increasing the absorption of antigen through some regions of nasal-associated lymphoid tissue^[^^33^^,^^34^^]^, an induction site for immune response in the upper respiratory tract^[^^35^^]^. The positive surface charge of chitosan and TMC nanoparticles raises the cellular interaction between the epithelial cells with a negative charge on their cell membrane and dendritic cells and thus increases the antigen residence time in the nasal cavity^[^^33^^,^^34^^]^. Since chitosan and its water-soluble derivative, TMC, are good nanocarriers for trans-mucosal vaccination especially nasal vaccine^[^^33^^,^^35^^]^, we used these biopolymers for nasal delivery of rEIT antigen. The production of chitosan and TMC nanoparticles was carried out using an electrospraying technique^[^^22^^]^. Electrospraying process was carried out at 20 kV as described by Doan *et a1.*^[^^26^^]^. A previous research has shown that a reduction in acetic acid concentration used for the preparation of chitosan solution results in lower viscosity of chitosan solution^[^^25^^]^. Therefore, we applied 70% (v/v) acetic acid as chitosan solvent.

To achieve the stability of particles suspension and the inhibition of the particles agglomeration, the zeta potential of particles would be in highly negative or positive values (lower than -30 mV or higher than +30 mV)^[^^36^^]^. The observed zeta potential values of resultant nanoparticles prepared here were above +30 mV, which resulted in stable nanoparticles (Fig. 2D). The nanoparticles size of 300-400 nm (Fig. 1) obtained in the present study was comparable to other studies^[^^20^^,^^26^^,^^37^^]^. In this continuous one-step process, we avoided the use of an external dispersion/emulsion phase because this issue might decrease the chance of its unpredictable side effects on rEIT protein^[22]^. Our data depicted that intranasal immunization with electrosprayed rEIT-chitosan or TMC nanoparticles significantly reduced *E. coli *O157:H7 shedding following the infection by 10^10^ CFU of live bacteria (Fig. 6). The earlier results indicated that intranasally immunized mice with a current nanovaccine formulation specially with TMC-adjuvanted nanovaccine offered more reduction in bacterial shedding period compared to intraperitoneally immunized mice (*P*<0.05) (Fig. 6). A significant (*P*<0.05) reduction was observed in *E. coli* O157:H7 attachment rate to a Caco-2 cell line with a serum of intranasally immunized mice as compared to non-immunized mice (Table 3). Our results indicated that *E. coli *O157:H7 intranasal vaccines induced efficient immune responses. 

The histological macroscopic observations of colon and caecum specimens in challenged mice showed that the number of detectable bacteria in these specimens from intranasally or intraperitoneally immunized mice was significantly less than those detected in non-immunized mice (Fig. 7B, 7C, 7D, and 7E). No adhered bacteria were found in epithelial smoothen surface in mice immunized intranasally with electrosprayed nanoparticles TMC-rEIT (Fig. 7C). Furthermore, the shape and weight of the specimens from immunized and non-immunized mice were clearly different (Fig. 7A). Also, the induction of IgA is not essential for the reduction of *E. coli *O157:H7 colonization in mice^[^^7^^]^. It has been shown that SIgA is important and beneficial for the prevention of *E. coli *O157:H7 colonization and achievement of more efficacious protection^[^^38^^]^. Inhibition of EspA, intimin, and Tir functions, as bacterial receptors, is related to the IgA production^[^^8^^]^. Previous studies on EHEC nasal vaccines have indicated that only nasal administered vaccines could induce detectable SIgA titers in contrast to other injectable vaccines^[^^12^^-^^14^^]^. 

In conclusion, the results suggest that intranasal nanovaccine is a promising and an effective method that can serve as an alternative strategy for vaccine delivery against the *E. coli *O157:H7. The present investigation shows that the recombinant combination of EspA, intimin, and Tir is a potent, trivalent immunogen for intranasal nanovaccine. The rEIT trivalent protein formulated with electrosprayed chitosan or TMC nanoparticles can protect host against *E. coli *O157:H7 as a result of strong humoral and mucosal immune responses. 
